# Supplementary material for: Validation of Serum Calprotectin Relative to Other Biomarkers of Infection in Febrile Infants Presenting to the Emergency Department
Source: Antibiotics (Basel). 2024 May 7;13(5):425. doi: 10.3390/antibiotics13050425 (PMC11117379; doi:10.3390/antibiotics13050425)
Supplement: Supplementary file 1 [file antibiotics-13-00425-s001.zip › antibiotics-2902058-supplementary.pdf]

Supplementary Materials

Table S1. Analytical information for serum calprotectin assay (GCAL®, Gentian AS) on an Abbott Architect instrument (The Hospital for Sick Children, Toronto, ON, Canada).

Characteristic	Information
Limit of Quantification (manufacturer defined)	0.30 mg/L
Limit of Detection (manufacturer defined)	0.15 mg/L
Analytical Measuring Range (manufacturer defined)	0.43-20.66 mg/L
Precision	Level 1 [mean (CV)]: 1.04 mg/L (7.5%)<br>Level 2 [mean (CV)]: 10.1 mg/L (1.5%)
Linearity	Range Evaluated: 0–19 mg/L<br>Slope: 0.97<br>Intercept: 0.12 mg/L<br>R^2: 0.999
Method Comparison	Range Evaluated: 0–19 mg/L<br>Slope: 0.9502<br>Intercept: -0.08<br>R^2: 0.997<br>Average Bias: -0.18 mg/L
